# Synthesis and crystal structure of *catena*-poly[cobalt(II)-di-μ-chlorido-μ-pyridazine-κ^2^ *N* ^1^:*N* ^2^]

**DOI:** 10.1107/S2056989023007065

**Published:** 2023-09-08

**Authors:** Christian Näther, Inke Jess

**Affiliations:** a Institut für Anorganische Chemie, Universität Kiel, Max-Eyth.-Str. 2, 24118 Kiel, Germany; University of Aberdeen, United Kingdom

**Keywords:** synthesis, crystal structure, one-dimensional coordination compound

## Abstract

The reaction of cobalt dichloride hexa­hydrate with pyridazine leads to the formation of crystals of the title compound, [CoCl_2_(C_4_H_4_N_2_)]_*n*_. This compound is isotypic to a number of compounds with other divalent metal ions. Its asymmetric unit consists of a Co^2+^ atom (site symmetry 2/*m*), a chloride ion (site symmetry *m*) and a pyridazine mol­ecule (all atoms with site symmetry *m*). The Co^2+^ cations are coordinated by four chloride anions and two pyridazine ligands, generating *trans*-CoN_4_Cl_2_ octa­hedra, and are linked into [010] chains by pairs of μ-1,1-bridging chloride anions and bridging pyridazine ligands. In the crystal structure, the pyridazine ligands of neighboring chains are stacked onto each other, indicating π–π inter­actions. Powder X-ray diffraction proves that a pure crystalline phase was obtained. Differential thermonalysis coupled to thermogravimetry (DTA–TG) reveal that decomposition is observed at about 710 K. Magnetic measurements indicate low-temperature metamagnetic behavior as already observed in a related compound.

## Abstract 2

In the crystal structure of the title compound, the cobalt cations are octa­hedrally coordinated by pairs of μ-1,1-bridging chloride anions and bridging pyridazine ligands and linked into chains propagating along the crystallographic *b*-axis direction.

## 1. Chemical context

Mono-periodic coordination polymers have always attracted much inter­est because of their versatile physical properties (Leong & Vittal, 2011 ▸; Mas-Ballesté *et al.*, 2010 ▸; Cernák *et al.*, 2002 ▸; Chen & Suslick, 1993 ▸; Khlobystov *et al.*, 2001 ▸). This includes mono-periodic coordination polymers, which can show a variety of different magnetic properties including single-chain magnetic behavior (Lescouëzec *et al.*, 2005 ▸; Rams *et al.*, 2020 ▸; Werner *et al.*, 2015 ▸; Sun *et al.*, 2010 ▸; Dhers *et al.*, 2015 ▸). In this context, of inter­est are coordination polymers based on transition-metal halides in which the metal cations are linked by pairs of μ-1,1-bridging halide anions into chains. The most prominent cations such as Mn^II^, Fe^II^, Co^II^ or Ni^II^ are mostly octa­hedrally coordinated, which means that for the synthesis of compounds with chain structures, mono-coordinating ligands must be used and several such compounds have already been reported in the literature (Foner *et al.*, 1975 ▸, 1978 ▸; Qin *et al.*, 2015 ▸; Zheng *et al.*, 2010 ▸). Compounds with a chain structure may also be observed if ligands such as tetra­zole or pyridazine derivatives are used, in which the nitro­gen donor atoms are adjacent. In this case, the chain structure remains unchanged and the co-ligand bridges two neighboring metal cations within the chain (Ivashkevich *et al.*, 2009 ▸; Masciocchi *et al.*, 1994 ▸; Thomas & Ramanan, 2016 ▸). In this context, Masciocchi and coworkers have reported on compounds with the composition Ni*X*_2_(C_4_H_4_N_2_) where C_4_H_4_N_2_ = pyridazine (1,2-diazine) with *X* = Cl, Br that were structurally characterized by X-ray powder diffraction (Masciocchi *et al.*, 1994 ▸). In these structures, the Ni^II^ cations are linked by pairs of halide anions into chains and within the chains, neighboring Ni^II^ cations are additionally bridged by the pyridazine ligands. 
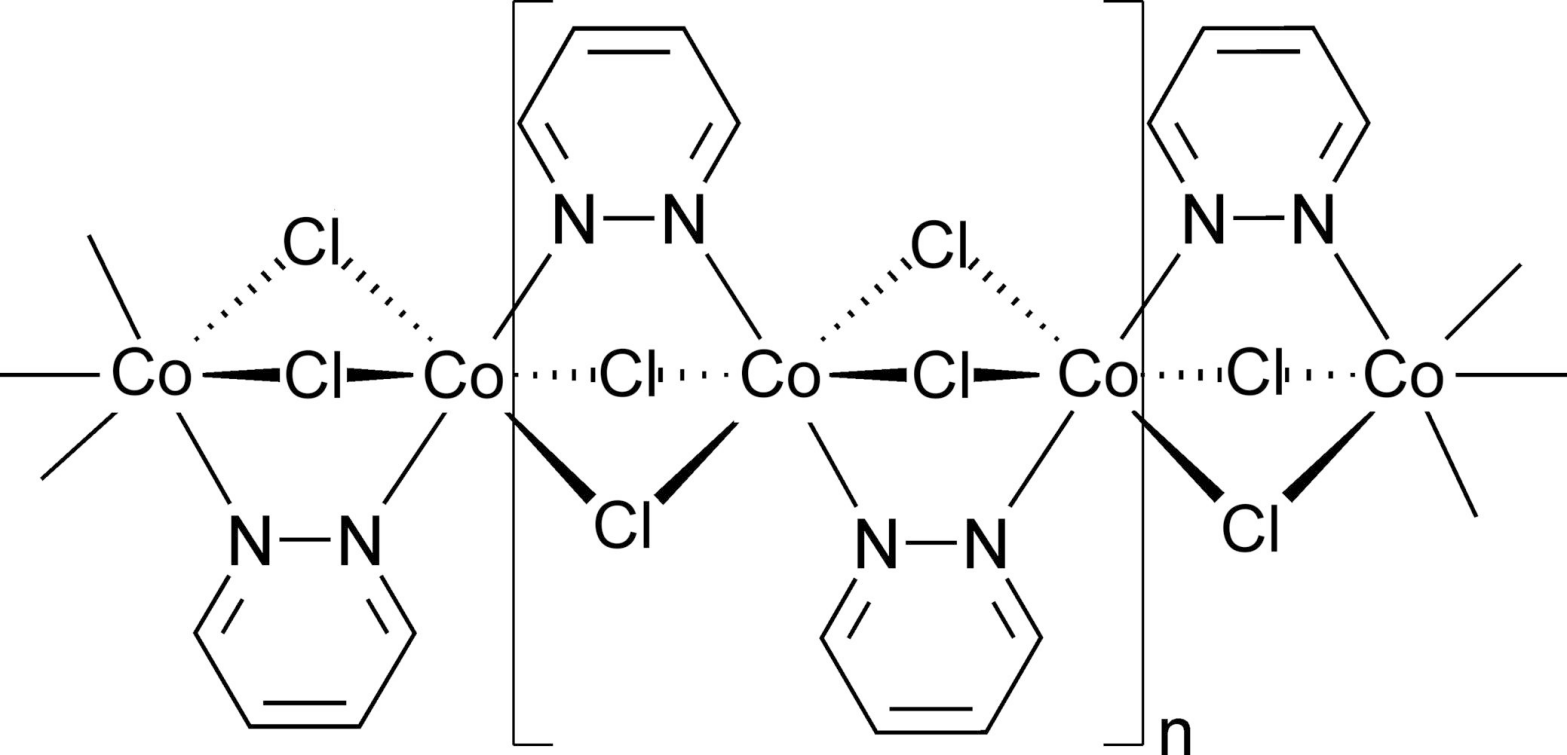


We are also inter­ested in coordination polymers in which the metal cations are linked by small-sized anionic ligands into one- or two-dimensional networks. In the beginning, we investigated compounds based on Cu^I^ cations and halide anions with additional N-donor coligands because we have found that, upon heating, they lose their coligands in a stepwise manner and transform into new coligand-deficient compounds that show condensed copper–halide networks (Näther & Jess, 2004 ▸; Näther *et al.*, 2001 ▸, 2007 ▸). Later we found that this synthetic procedure can also be used for compounds with divalent cations such as Cd^II^ (Näther *et al.*, 2017 ▸). In the course of this project, we also became inter­ested in metal-halide compounds with paramagnetic metal cations and as part of these investigations, we reacted CoCl_2_ with pyridazine and obtained a compound with the composition CoCl_2_(C_4_H_4_N_2_). No entry was found in the Cambridge Structural Database (CSD, version 5.43, last update March 2023; Groom *et al.*, 2016 ▸) and therefore this compound was characterized by single-crystal X-ray diffraction. Later we found that this compound had already been characterized by X-ray powder diffraction and it was concluded that it is isotypic to its Ni^II^ counterpart (Masciocchi *et al.*, 1994 ▸), which we found is the case.

## 2. Structural commentary

**Figure 1:**
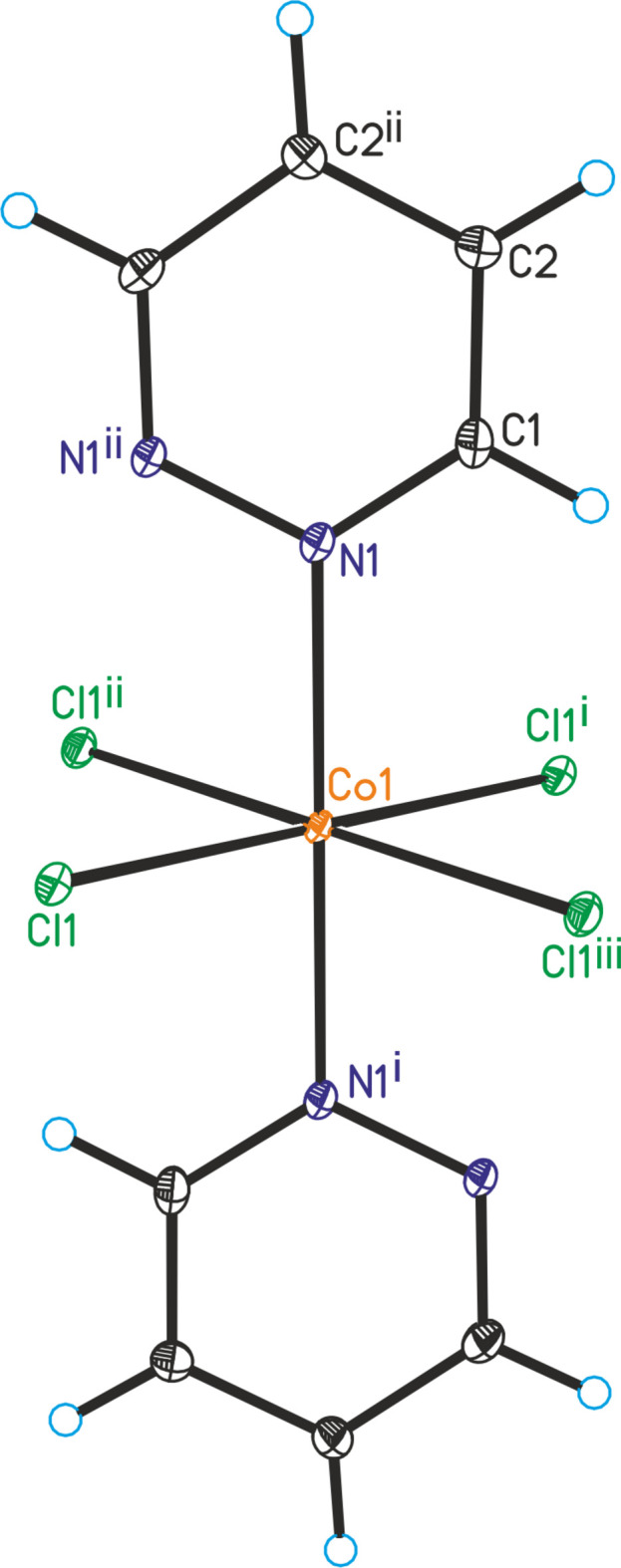
Crystal structure of the title compound with labeling and displacement ellipsoids drawn at the 50% probability level. Symmetry codes for the generation of equivalent atoms: (i) −*x* + 1, 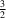 − *y*, *z*; (ii) −*x* + 1, −*y* + 1, −*z* + 1; (iii) *x*, − 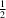 + *y*, −*z* + 1.

**Figure 2:**
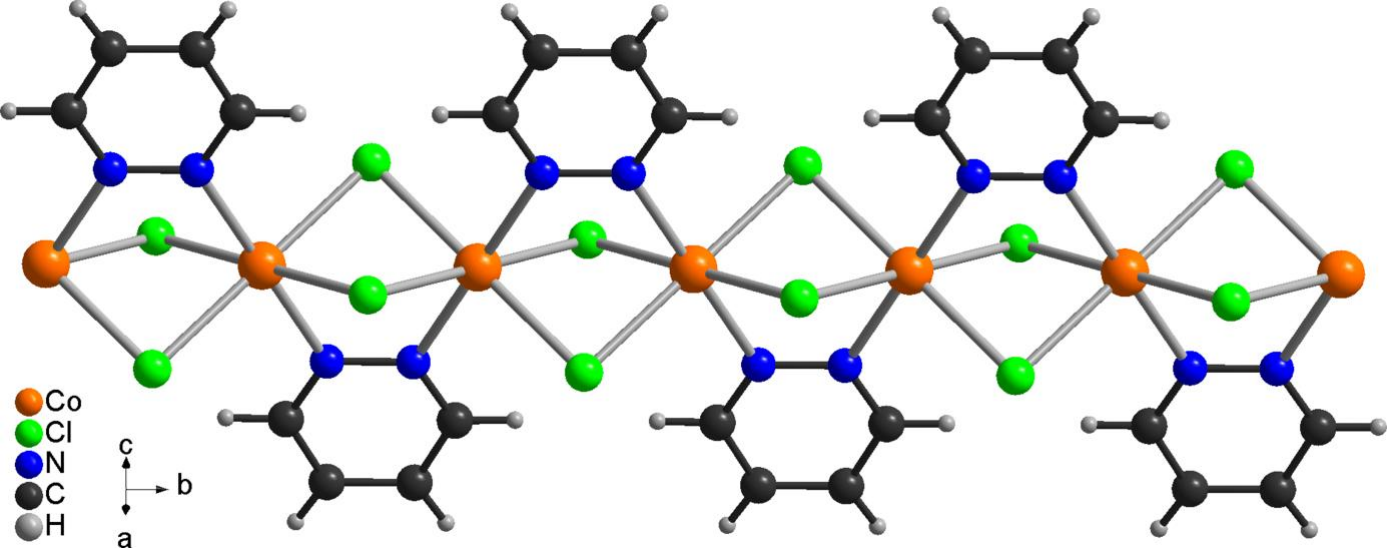
Fragment of a [010] polymeric chain in the title compound.

The reaction of CoCl_2_·6H_2_O with pyridazine in water in a sealed vessel at 388 K leads to the formation of single crystals of the title compound CoCl_2_(C_4_H_4_N_2_). This compound is isotypic to its Mn^II^, Fe^II^ and Ni^II^ analogs with chloride and bromide as counter-anions, already reported in the literature (Masciocchi *et al.*, 1994 ▸). The asymmetric unit consists of one cobalt(II) cation located at (1/4, 1/4, 1/4) on the inter­section point of a twofold screw axis and a mirror plane (Wyckoff site 4*c*, site symmetry 2/*m*), as well as one chloride anion at (1/2, *y*, *z*) that is situated on a mirror plane on Wyckoff site 8*h*. The asymmetric unit also contains half a pyridazine ligand with all atoms located at (*x*, 1/4, *z*) on Wyckoff position 8*i* (*m* site symmetry): the complete C_4_H_4_N_2_ ligand is generated by a second mirror plane at *x* = 1/2 (Fig. 1 ▸). The Co^II^ cations are octa­hedrally coordinated by four chloride anions and two pyridazine ligands and from the bond lengths and angles, it is obvious that the octa­hedra are slightly distorted (Table 1 ▸). The Co^II^ cations are linked by pairs of μ-1,1-bridging chloride anions into chains that propagate in the *b*-axis direction (Fig. 2 ▸). The pyridazine ligands also act as bridging ligands, each connecting two neighboring Co^II^ cations. Within the chains, all of the pyridazine ligands are coplanar. The intra­chain Co⋯Co distance is 3.3443 (3) Å.

## 3. Supra­molecular features

**Figure 3:**
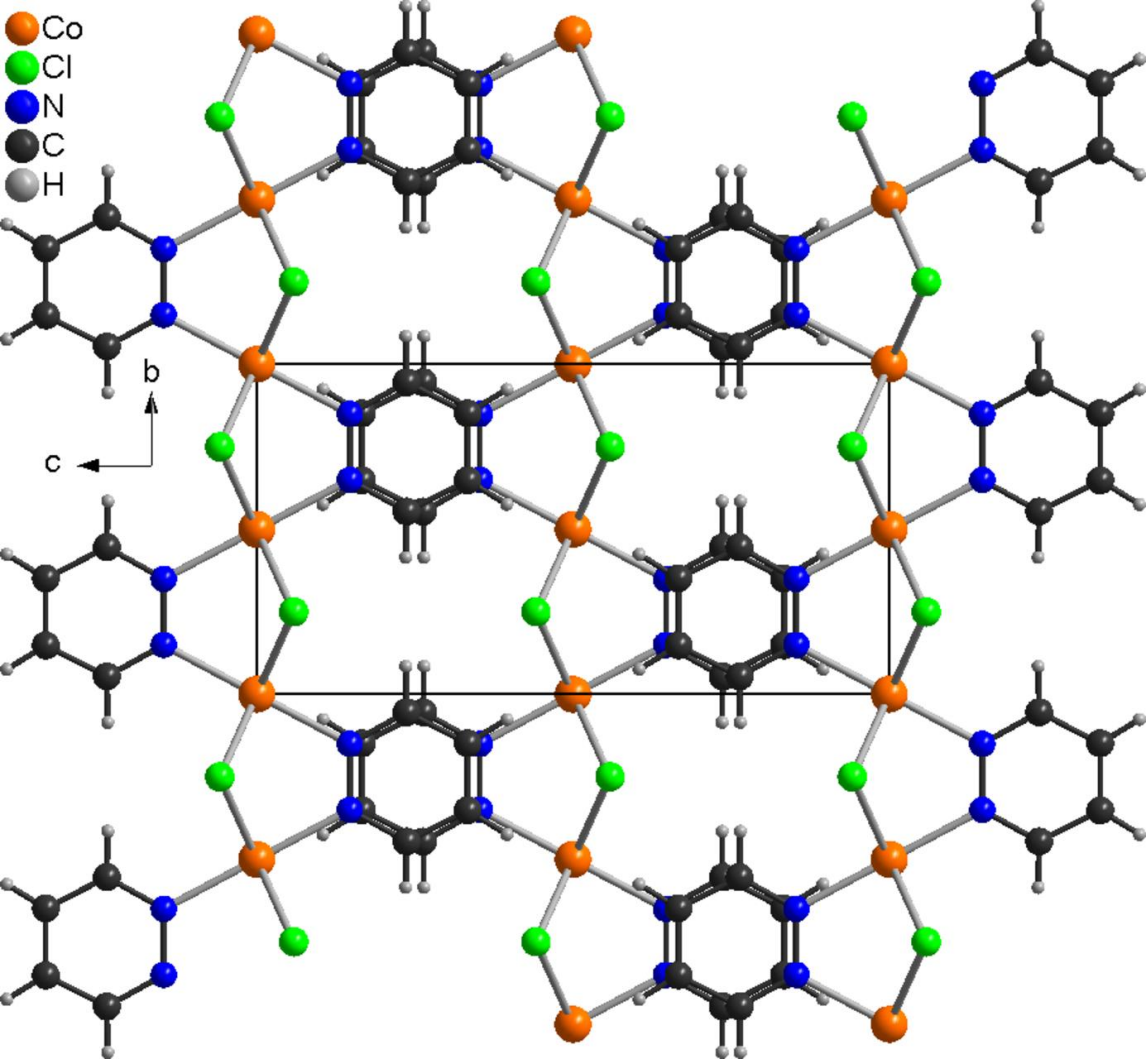
Arrangement of the chains in the crystal structure of the title compound with view along the crystallographic *a*-axis direction.

In the crystal structure of the title compound, the chains propagate in the *b*-axis direction and are arranged in such a way that neighboring pyridazine ligands are perfectly stacked onto each other, forming columns along the crystallographic *a* axis (Fig. 3 ▸). The angle between two neighboring pyridazine ligands is 180° and the distance between their centroids is 3.6109 (1) Å (slippage = 0.264 Å), indicating π–π stacking inter­actions. One very weak C—H⋯Cl hydrogen bond (Table 2 ▸) is observed.

## 4. Database survey

Many compounds of the general formula *MX*_2_(C_4_H_4_N_2_) (*M* = transition metal and *X* = halide anion have already been reported in the Cambridge Structural Database but there are no hits for cobalt. The compounds with NiCl_2_ (CSD refcode POPCIG) and NiBr_2_ (POPCOM) were structurally characterized by Rietveld refinements using laboratory X-ray powder diffraction data (Masciocchi *et al.*, 1994 ▸). In this contribution, the compounds with Mn, Fe, Co, Cu and Zn with chloride and bromide as anions were also synthesized and from their powder patterns, the lattice parameters were determined, which indicate that the compounds with Mn, Fe and Co are isotypic to the Ni compounds; this is not the case for the compounds with Cu and Zn (Masciocchi *et al.*, 1994 ▸). Our determination definitively proves that the title compound is isotypic to its Ni analog. The compounds *M*Cl_2_(C_4_H_4_N_2_) with Mn (LANJEQ), Fe (LANJAM) were later determined by single-crystal X-ray diffraction, and their magnetic properties were also investigated (Yi *et al.*, 2002 ▸).

With copper, additional compounds were investigated by single-crystal X-ray diffraction, including CuCl_2_(C_4_H_4_N_2_) (JEFFOS; Thomas & Ramanan, 2016 ▸) and CuBr_2_(pyridiazine) (JEFFUY; Thomas & Ramanan, 2016 ▸). However, most compounds are reported with Cu^I^, including CuI(C_4_H_4_N_2_) (CAQXAT; Kromp & Sheldrick, 1999 ▸, and CAQXAT01; Thomas & Ramanan, 2016 ▸), CuBr(C_4_H_4_N_2_) (CAQXEX; Kromp & Sheldrick, 1999 ▸, and CAQXEX01 and 02; Thomas & Ramanan, 2016 ▸), Cu_2_I_2_(C_4_H_4_N_2_) (CAQXIB; Kromp & Sheldrick, 1999 ▸), Cu_2_Cl_2_(C_4_H_4_N_2_) (CAQXOH; Kromp & Sheldrick, 1999 ▸, and CAQXOH01 and 02; Thomas & Ramanan, 2016 ▸), two modifications of CuCl(C_4_H_4_N_2_) (EKINOB and EKINUH; Näther & Jess, 2003 ▸, and EKINUH01; Thomas & Ramanan, 2016 ▸), Cu_2_Br_2_(C_4_H_4_N_2_) (EKIPAP; Näther & Jess, 2003 ▸, and EKIPAP01; Thomas & Ramanan, 2016 ▸).

With diamagnetic Zn^II^, three compounds are reported, namely ZnI_2_(C_4_H_4_N_2_)_2_ (MENSUU; Bhosekar *et al.*, 2006*a* ▸), ZnBr_2_(C_4_H_4_N_2_)_2_ (VEMBEV; Bhosekar *et al.*, 2006*b* ▸) and three modifications of ZnCl_2_(C_4_H_4_N_2_)_2_ (YAFYOU, YAFYOU01, YAFYOU02 and YAFYOU03; Pazderski *et al.*, 2004*a* ▸ and Bhosekar *et al.*, 2007 ▸). Finally, the Cd compounds CdCl_2_(C_4_H_4_N_2_) (AZABUY; Pazderski *et al.*, 2004*b* ▸), CdBr_2_(C_4_H_4_N_2_) and CdI_2_(C_4_H_4_N_2_) have also been reported (refcodes to be assigned; Näther & Jess, 2023 ▸).

## 5. Physical characterization

The experimental powder pattern of the title compound agrees closely with that calculated from the single crystal data, which proves that a pure compound was obtained (Fig. S1). The thermal properties were investigated by differential thermoanalysis and thermogravimetry (DTA–TG) under an air atmosphere. Upon heating, only one mass loss is observed, which is accompanied by an endothermic event in the DTA curve (Fig. S2). The experimental mass loss of 27.4% is much lower than that calculated for the removal of one pyridazine ligand (38.2%), indicating that the pyridazine ligands are not completely removed. This is supported by the fact that the TG curve still decreases upon further heating. In the DTA curve, a successive endothermic and exothermic event is observed, which points to the decomposition of this compound (Fig. S2).

The title compounds were also characterized by magnetic measurements. The temperature dependence of the susceptibility was measured in the range 2–300 K under an applied magnetic field of 1000 Oe. Upon cooling, a maximum is observed at 3.0 K, indicating an anti­ferromagnetic transition (Fig. S3). The data were analyzed using a Curie–Weiss law, leading to a magnetic moment of 5.0 µ_B_, which is higher than expected for a Co^II^ cation in a high-spin 3*d* ^7^ configuration. The Weiss constant of −8 K suggests predominant anti­ferromagnetic inter­actions, but it must be kept in mind that these values are frequently too high because of the strong spin–orbit coupling of Co^II^. Additional field-dependent measurements at 2 K indicate metamagnetic behavior with no saturation even at high fields, as previously observed for the linear chain compound CoCl_2_(C_4_H_4_N_2_)_2_ (Fig. S4; Foner *et al.*, 1975 ▸).

## 6. Synthesis and crystallization

**Synthesis**

CoCl_2_·6H_2_O and pyridazine were purchased from Sigma Aldrich and pyridazine from Alfa Aesar. All chemicals were used without further purification.

Pink-colored crystals were obtained by the reaction of 1 mmol of Co(NCS)_2_ (237.9 mg) and 1 mmol (72 µl) of pyrid­azine in 1 ml of demineralized water. The reaction mixture was heated in a sealed glass vessel at 388 K for 2 d, leading to the formation of crystals suitable for single-crystal X-ray analysis. An IR spectrum of the title compound can be found in Fig. S5.

**Experimental details**

The IR spectrum was measured using an ATI Mattson Genesis Series FTIR Spectrometer, control software: WINFIRST, from ATI Mattson.

The PXRD measurement was performed with Cu *K*α_1_ radiation (λ = 1.540598 Å) using a Stoe Transmission Powder Diffraction System (STADI P) equipped with a MYTHEN 1K detector and a Johansson-type Ge(111) monochromator.

Thermogravimetry and differential thermoanalysis (TG–DTA) measurements were performed in a dynamic air atmosphere in Al_2_O_3_ crucibles using a STA-PT 1000 thermobalance from Linseis. The instrument was calibrated using standard reference materials.

Magnetic measurements were performed using a Quantum Design PPMS equipped with a 7 T magnet, using samples mounted in a gelatine capsule.

## 7. Refinement

Crystal data, data collection and structure refinement details are summarized in Table 3 ▸. The C—H hydrogen atoms were positioned with idealized geometry and refined as riding atoms with *U*_iso_(H) = 1.2 *U*_eq_(C).

## Supplementary Material

Crystal structure: contains datablock(s) I. DOI: 10.1107/S2056989023007065/hb8073sup1.cif

Structure factors: contains datablock(s) I. DOI: 10.1107/S2056989023007065/hb8073Isup2.hkl

Fig. S1. Experimental (top) and calculated (bottom) powder pattern of the title compound. DOI: 10.1107/S2056989023007065/hb8073sup3.png

Fig. S2. DTG (top), TG (middle) and DTA curve (bottom) for the title compound measured with 8C in air atmosphere. DOI: 10.1107/S2056989023007065/hb8073sup4.png

Fig. S3. Magnetic susceptibility as function of temperature measured for the title compound at 1000 Oe. DOI: 10.1107/S2056989023007065/hb8073sup5.png

Fig. S4. Field dependence of the magnetization measured for the title compound at 2 K. DOI: 10.1107/S2056989023007065/hb8073sup6.png

Fig. S5. IR spectrum of the title compound. The wave numbers of the most prominent vibrations are given. DOI: 10.1107/S2056989023007065/hb8073sup7.png

CCDC reference: 2287781

Additional supporting information:  crystallographic information; 3D view; checkCIF report

## Figures and Tables

**Table 1:** Selected geometric parameters (Å, °)

Co1—N1	2.1282 (19)	Co1—Cl1	2.4626 (4)
			
N1—Co1—N1^i^	180.0	Cl1—Co1—Cl1^i^	180.0
N1—Co1—Cl1	87.21 (4)	Cl1—Co1—Cl1^ii^	83.66 (2)
N1^i^—Co1—Cl1	92.79 (4)	Cl1^i^—Co1—Cl1^ii^	96.34 (2)

Symmetry codes: (i) 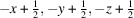 ; (ii) 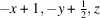 .

**Table 2:** Hydrogen-bond geometry (Å, °)

*D*—H⋯*A*	*D*—H	H⋯*A*	*D*⋯*A*	*D*—H⋯*A*
C2—H2⋯Cl1^iii^	0.95	2.97	3.574 (2)	123

Symmetry code: (iii) 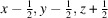 .

**Table 3:** Experimental details

Crystal data	
Chemical formula	[CoCl_2_(C_4_H_4_N_2_)]
*M*_r_	209.92
Crystal system, space group	Orthorhombic, *I* *m* *m* *a*
Temperature (K)	100
*a*, *b*, *c* (Å)	6.6935 (1), 7.2024 (1), 12.7978 (2)
*V* (Å^3^)	616.97 (2)
*Z*	4
Radiation type	Cu *K*α
μ (mm^−1^)	28.91
Crystal size (mm)	0.1 × 0.08 × 0.08

Data collection	
Diffractometer	XtaLAB Synergy, Dualflex, HyPix
Absorption correction	Multi-scan (*CrysAlis PRO*; Rigaku OD, 2022 ▸)
*T*_min_, *T*_max_	0.316, 1.000
No. of measured, independent and observed [*I* > 2σ(*I*)] reflections	3164, 395, 386
*R*_int_	0.027
(sin θ/λ)_max_ (Å^−1^)	0.639

Refinement	
*R*[*F* ^2^ > 2σ(*F* ^2^)], *wR*(*F* ^2^), *S*	0.020, 0.058, 1.07
No. of reflections	395
No. of parameters	30
H-atom treatment	H-atom parameters constrained
Δρ_max_, Δρ_min_ (e Å^−3^)	0.64, −0.34

Computer programs: *CrysAlis PRO* (Rigaku OD, 2022 ▸), *SHELXT2014* (Sheldrick, 2015*a* ▸), *SHELXL2016* (Sheldrick, 2015*b* ▸), *DIAMOND* (Brandenburg, 1999 ▸) and *publCIF* (Westrip, 2010 ▸).

## supplementary crystallographic information

### Crystal data

**Table:**

[CoCl_2_(C_4_H_4_N_2_)]	*D*_x_ = 2.260 Mg m^−^^3^
*M**_r_* = 209.92	Cu *K*α radiation, λ = 1.54184 Å
Orthorhombic, *I**m**m**a*	Cell parameters from 2503 reflections
*a* = 6.6935 (1) Å	θ = 6.9–78.2°
*b* = 7.2024 (1) Å	µ = 28.91 mm^−^^1^
*c* = 12.7978 (2) Å	*T* = 100 K
*V* = 616.97 (2) Å^3^	Block, pink
*Z* = 4	0.1 × 0.08 × 0.08 mm
*F*(000) = 412	

### Data collection

**Table:**

XtaLAB Synergy, Dualflex, HyPix diffractometer	395 independent reflections
Radiation source: micro-focus sealed X-ray tube, PhotonJet (Cu) X-ray Source	386 reflections with *I* > 2σ(*I*)
Mirror monochromator	*R*_int_ = 0.027
Detector resolution: 10.0000 pixels mm^-1^	θ_max_ = 80.2°, θ_min_ = 6.9°
ω scans	*h* = −8→8
Absorption correction: multi-scan (CrysAlis PRO; Rigaku OD, 2022)	*k* = −8→8
*T*_min_ = 0.316, *T*_max_ = 1.000	*l* = −16→15
3164 measured reflections	

### Refinement

**Table:**

Refinement on *F*^2^	Hydrogen site location: inferred from neighbouring sites
Least-squares matrix: full	H-atom parameters constrained
*R*[*F*^2^ > 2σ(*F*^2^)] = 0.020	*w* = 1/[σ^2^(*F*_o_^2^) + (0.039*P*)^2^ + 0.9618*P*] where *P* = (*F*_o_^2^ + 2*F*_c_^2^)/3
*wR*(*F*^2^) = 0.058	(Δ/σ)_max_ < 0.001
*S* = 1.07	Δρ_max_ = 0.64 e Å^−^^3^
395 reflections	Δρ_min_ = −0.34 e Å^−^^3^
30 parameters	Extinction correction: SHELXL2016 (Sheldrick, 2015b), Fc^*^=kFc[1+0.001xFc^2^λ^3^/sin(2θ)]^-1/4^
0 restraints	Extinction coefficient: 0.0016 (3)
Primary atom site location: dual	

### Special details

**Table:**

Geometry. All esds (except the esd in the dihedral angle between two l.s. planes) are estimated using the full covariance matrix. The cell esds are taken into account individually in the estimation of esds in distances, angles and torsion angles; correlations between esds in cell parameters are only used when they are defined by crystal symmetry. An approximate (isotropic) treatment of cell esds is used for estimating esds involving l.s. planes.

### Fractional atomic coordinates and isotropic or equivalent isotropic displacement parameters (Å^2^)

**Table:**

	*x*	*y*	*z*	*U*_iso_*/*U*_eq_	
Co1	0.250000	0.250000	0.250000	0.0069 (2)	
Cl1	0.500000	0.47804 (8)	0.19118 (4)	0.0089 (2)	
N1	0.3991 (3)	0.250000	0.39687 (15)	0.0082 (4)	
C1	0.3025 (4)	0.250000	0.48765 (19)	0.0126 (5)	
H1	0.160605	0.250000	0.486369	0.015*	
C2	0.3973 (4)	0.250000	0.58452 (17)	0.0112 (5)	
H2	0.323309	0.250000	0.647854	0.013*	

### Atomic displacement parameters (Å^2^)

**Table:**

	*U*^11^	*U*^22^	*U*^33^	*U*^12^	*U*^13^	*U*^23^
Co1	0.0036 (3)	0.0111 (3)	0.0062 (3)	0.000	−0.00030 (17)	0.000
Cl1	0.0060 (3)	0.0108 (3)	0.0099 (3)	0.000	0.000	0.00073 (17)
N1	0.0052 (9)	0.0107 (9)	0.0087 (8)	0.000	0.0001 (7)	0.000
C1	0.0076 (11)	0.0184 (12)	0.0118 (11)	0.000	0.0020 (10)	0.000
C2	0.0103 (12)	0.0142 (11)	0.0090 (11)	0.000	−0.0005 (9)	0.000

### Geometric parameters (Å, º)

**Table:**

Co1—N1	2.1282 (19)	N1—C1	1.330 (3)
Co1—N1^i^	2.1282 (19)	N1—N1^ii^	1.350 (4)
Co1—Cl1	2.4626 (4)	C1—C2	1.393 (3)
Co1—Cl1^i^	2.4626 (4)	C1—H1	0.9500
Co1—Cl1^ii^	2.4626 (4)	C2—C2^ii^	1.374 (5)
Co1—Cl1^iii^	2.4626 (4)	C2—H2	0.9500
			
N1—Co1—N1^i^	180.0	Cl1^i^—Co1—Cl1^iii^	83.66 (2)
N1—Co1—Cl1	87.21 (4)	Cl1^ii^—Co1—Cl1^iii^	180.0
N1^i^—Co1—Cl1	92.79 (4)	Co1—Cl1—Co1^ii^	85.611 (17)
N1—Co1—Cl1^i^	92.79 (4)	C1—N1—N1^ii^	119.10 (15)
N1^i^—Co1—Cl1^i^	87.21 (4)	C1—N1—Co1	122.93 (17)
Cl1—Co1—Cl1^i^	180.0	N1^ii^—N1—Co1	117.97 (5)
N1—Co1—Cl1^ii^	87.21 (4)	N1—C1—C2	123.8 (3)
N1^i^—Co1—Cl1^ii^	92.79 (4)	N1—C1—H1	118.1
Cl1—Co1—Cl1^ii^	83.66 (2)	C2—C1—H1	118.1
Cl1^i^—Co1—Cl1^ii^	96.34 (2)	C2^ii^—C2—C1	117.11 (16)
N1—Co1—Cl1^iii^	92.79 (4)	C2^ii^—C2—H2	121.4
N1^i^—Co1—Cl1^iii^	87.21 (4)	C1—C2—H2	121.4
Cl1—Co1—Cl1^iii^	96.34 (2)		

Symmetry codes: (i) −*x*+1/2, −*y*+1/2, −*z*+1/2; (ii) −*x*+1, −*y*+1/2, *z*; (iii) *x*−1/2, *y*, −*z*+1/2.

### Hydrogen-bond geometry (Å, º)

**Table:**

*D*—H···*A*	*D*—H	H···*A*	*D*···*A*	*D*—H···*A*
C2—H2···Cl1^iv^	0.95	2.97	3.574 (2)	123

Symmetry code: (iv) *x*−1/2, *y*−1/2, *z*+1/2.
