# Supplementary figures and images for: Synthesis and crystal structure of catena-poly[cobalt(II)-di-μ-chlorido-μ-pyridazine-κ2 N 1:N 2]
Source: Acta Crystallogr E Crystallogr Commun. 2023 Sep 8;79(Pt 10):872–6. doi: 10.1107/S2056989023007065 (PMC10561210; doi:10.1107/S2056989023007065)

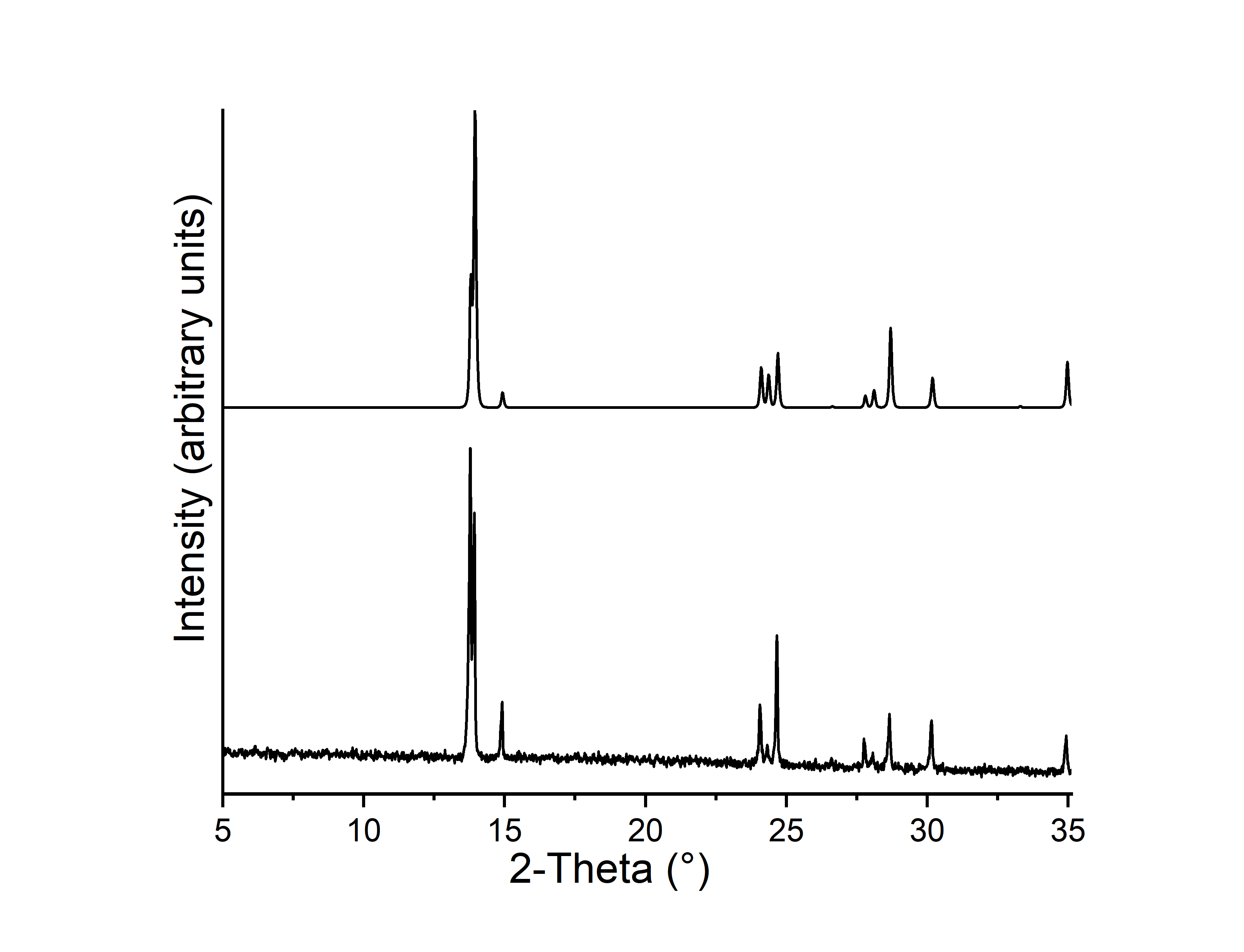

Supplement: Supplementary file 3 [file e-79-00872-sup3.png]

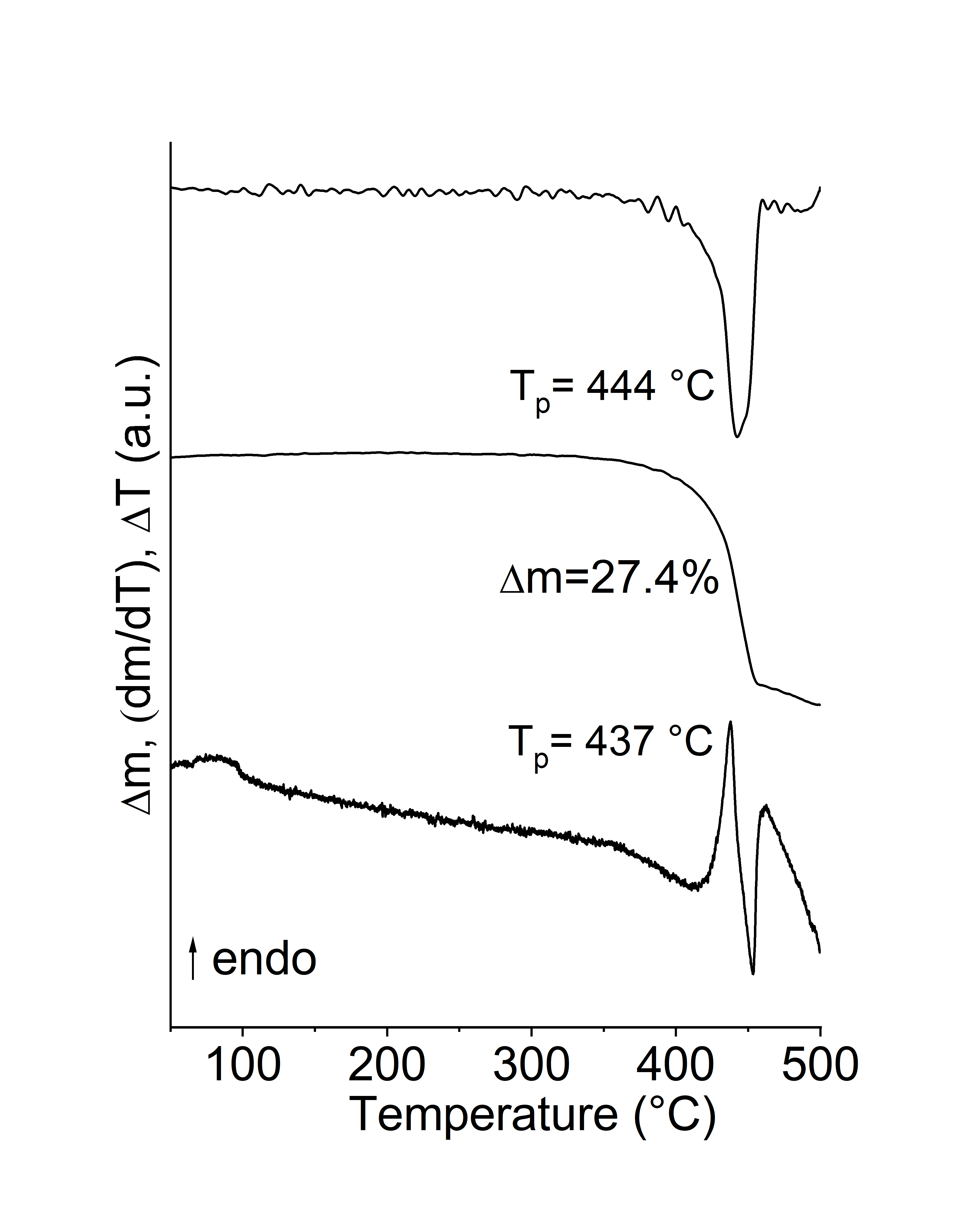

Supplement: Supplementary file 4 [file e-79-00872-sup4.png]

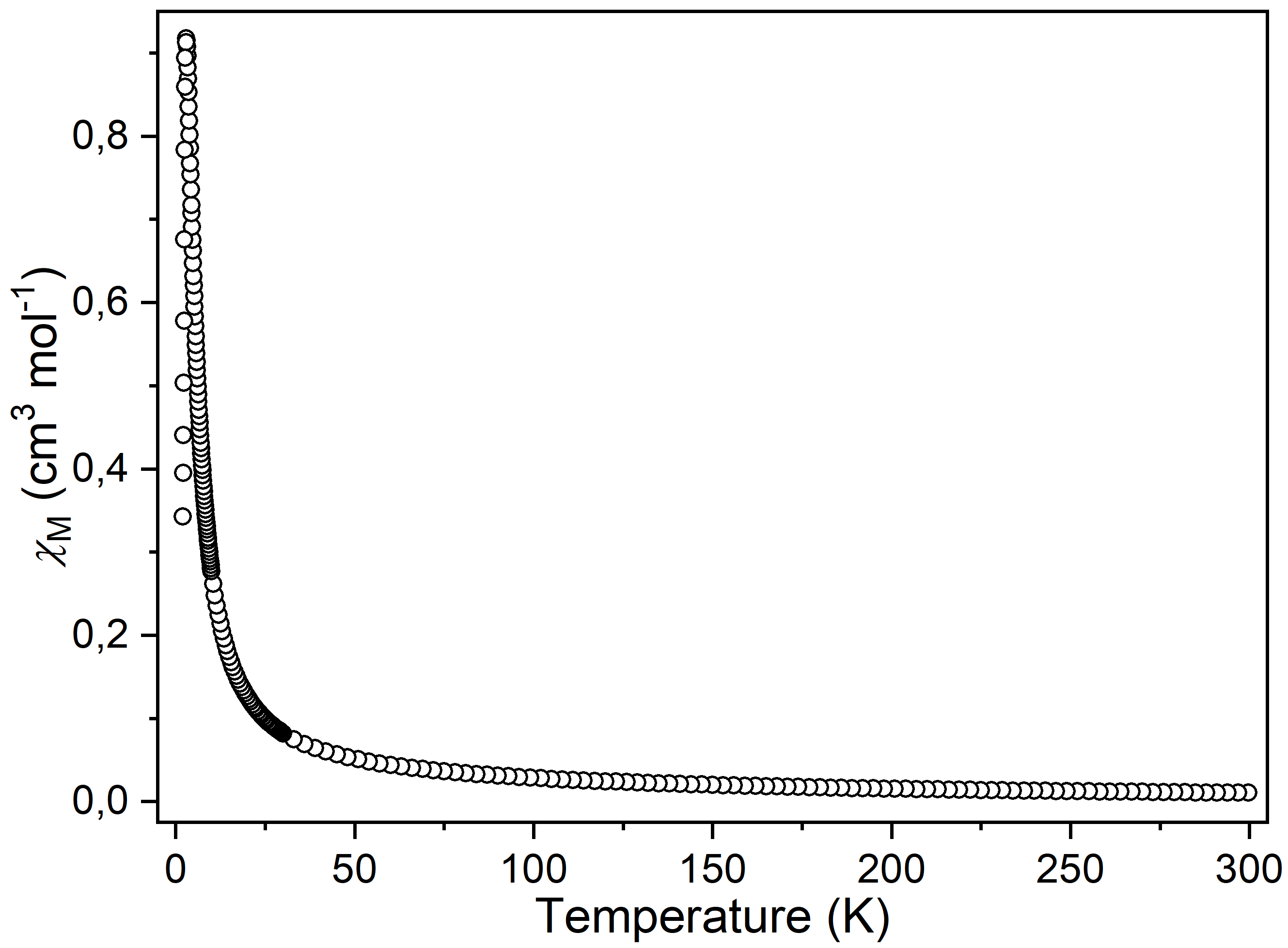

Supplement: Supplementary file 5 [file e-79-00872-sup5.png]

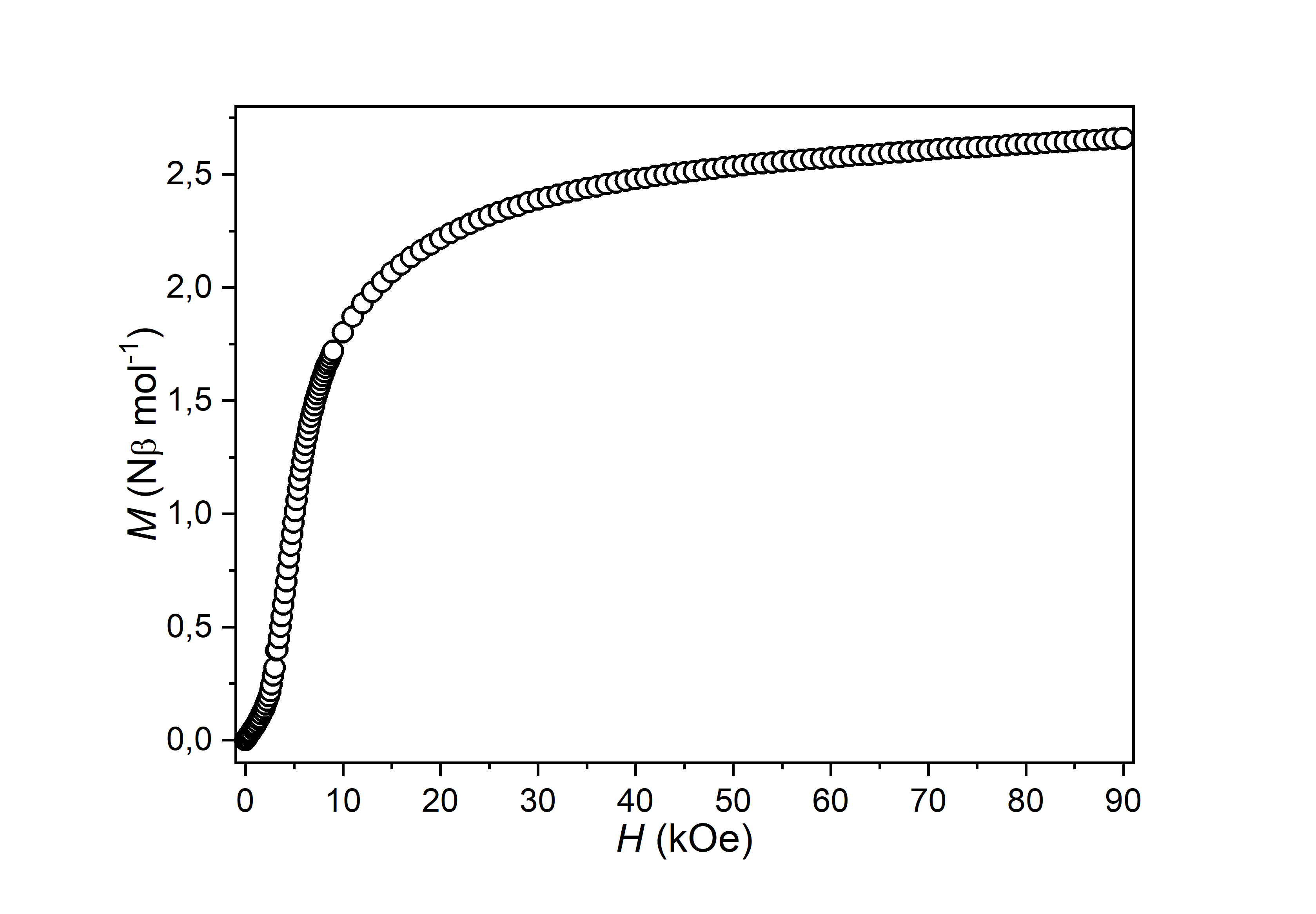

Supplement: Supplementary file 6 [file e-79-00872-sup6.png]

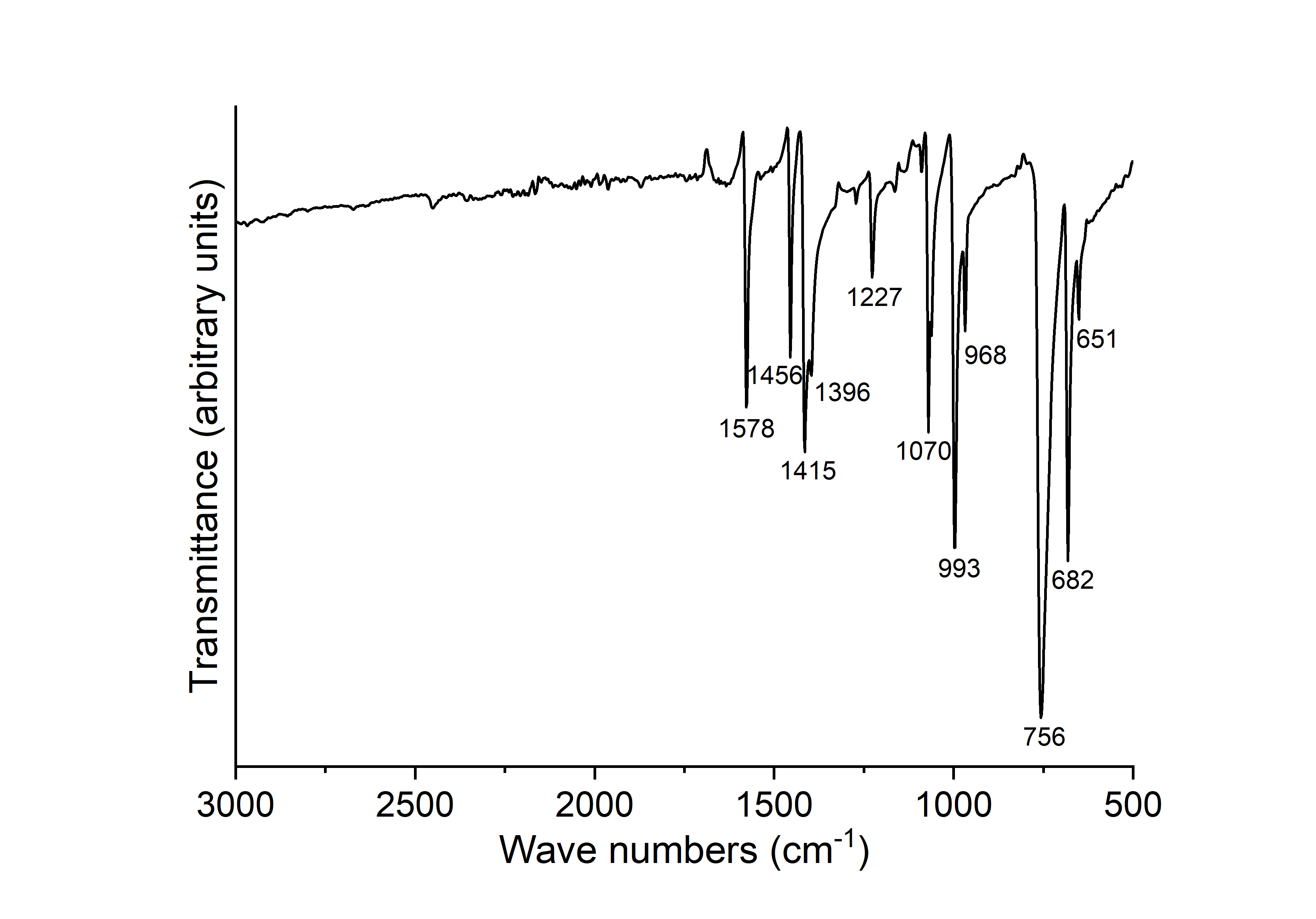

Supplement: Supplementary file 7 [file e-79-00872-sup7.png]
